# Will the white blood cells tell? A potential novel tool to assess broiler chicken welfare

**DOI:** 10.3389/fvets.2024.1384802

**Published:** 2024-07-02

**Authors:** Laura Raquel Rios Ribeiro, Elaine Cristina de Oliveira Sans, Ricardo Martins Santos, Cesar Augusto Taconelli, Roberta de Farias, Carla Forte Maiolino Molento

**Affiliations:** ^1^Animal Welfare Laboratory, Department of Animal Science, Federal University of Paraná, Curitiba, Brazil; ^2^National Animal Defense Forum, Curitiba, Brazil; ^3^Immunology Laboratory, Dom Bosco Catholic University, Campo Grande, Brazil; ^4^Department of Statistics, Federal University of Paraná, Curitiba, Brazil

**Keywords:** animal welfare, hematology, heterophil, inflammation, leukocyte, lymphocytes

## Abstract

This study assessed qualitative and quantitative leukocyte evaluation as potential broiler chicken welfare indicators, contributing to the limited literature on white blood cell (WBC) morphology as a diagnostic tool for welfare. Broiler chicken welfare within four poultry houses (PH) 1 to 4, each on a different farm, was assessed using on-field indicators of affective states and health, and WBC morphology was examined. Affective states were evaluated using the Qualitative Behavior Assessment (QBA), with 25 behavioral expressions scored on a visual analogue scale (VAS) and grouped into two categories. Health indicators included assessments of lameness, footpad dermatitis, dermatitis on the breast and abdominal areas, hock burn, and feather cleaning. Blood samples were collected, differential leukocyte counts were performed, and a cell score was created for the recognition, classification, and interpretation of morphologic diversity of heterophils and lymphocytes. The heterophil to lymphocyte ratio (H/L) was also determined. Descriptive statistics and generalized linear models for binomial responses were used to analyze the results. PH4 differed from the other farms, showing a higher frequency of birds within QBA group 1 (‘Attentive’to ‘Desperate’), while birds in PH1, PH2, and PH3 were more frequent in QBA group 2 (‘Relaxed’ to ‘Positively occupied’). Elevated proportions of heterophils in birds from PH4 (0.61, CI95%: 0.58; 0.64) and PH3 (0.60, CI95%: 0.57; 0.63) suggested higher stress levels and inflammatory responses. Birds in PH2 and PH4 exhibited higher frequencies of health issues such as dermatitis and lameness, and higher proportions of abnormalities in WBC number and morphology. PH3 and PH4 exhibited higher H/L ratios of 3.03 and 2.58, respectively, consistent with the on-field health and behavioral indicators. Blood samples from birds in PH2 and PH4 showed a proportion of 90% toxic change in heterophils, while in PH1 and PH3 it was 70%, indicating high levels of abnormal WBC morphology across all PHs. The findings emphasize the multifactorial nature of welfare impairments, including environmental conditions, health, and affective states. This highlights the need for indicators that reflect multiple welfare impacts, such as WBC counts and morphological alterations, which can serve as powerful tools in the complex task of assessing animal welfare.

## 1 Introduction

Brazil’s food production is among the largest in the world. Poultry production ranks first globally among all the animal species used for food production. Brazil is one of the world’s leading chicken producers and exporters, while the state of Parana is the largest chicken meat producer and exporter in Brazil, representing more than 40% of Brazilian exports. The Brazilian Animal Protein Association (ABPA) reported that Brazilian chicken meat exports, including both fresh and processed products, reached 3.90 million tons between January and September 2023, which is 6.5% higher than the total exported in the same period in 2022, with 3.66 million tons. In total, Brazil produced 14.90 million tons of chicken meat in 2023 and will produce up to 15.30 million tons in 2024 (1). Thus, due to the large number of individuals involved (2–4), poultry production may be considered a priority in animal welfare initiatives in Brazil.

Animal welfare (AW) enhancement can be driven by many factors, such as consumer and market demands, corporate interests, the implementation of new policies, the availability of funding, government incentives, country and regional nuances, climatic conditions, sustainability, and reduced carbon footprint and specific on-farm details such as housing design, management practices, and staff training (5, 6). For decades, concerns for AW expressed by consumers and evolving human attitudes toward animals have led to discussion on potential global policies for farm AW (7). In addition, for animal production systems to continue, they will depend on the ethical nature and sustainability of farming methods (2). For example, breeding and management conditions, producer’s attitude toward his or her animals, nutrition, the environment, health, behavior, and intensive feeding systems represent some of the factors that can negatively affect AW (8). As such, they may also affect public opinion in relation to the acceptability of broiler chicken intensive farming. However, proportionate public reactions and the development of adequate policies are only possible when appropriate AW assessment is available. Indeed, for all aspects of efforts toward AW improvement, reliable assessment tools are essential.

Animal welfare assessment is a multidisciplinary field that involves several measures and combinations of physiological and behavioral indicators, along with the application of specific protocols, many of which focus on different aspects of the animal’s life (9); Welfare (10). Physiological indicators, such as blood glucocorticoid levels, body temperature and leukocyte count, provide valuable information about the stress and general health of the animals. In addition, behavior measurements, such as the behavioral restrictions which are frequently imposed to farm animals and the expression of signs of discomfort or abnormal social interactions for the species, play a crucial role in assessing AW (11–16).

Most farm AW assessment protocols were initially devised for non-tropical farming characteristics; consequently, assessment protocols specifically designed for Brazilian animal production systems, considering the environmental, social, and economic factors in the country, are required. Importing AW standards based on such protocols is also complex, resulting in situations where compliance to AW standards may not improve the lives of the animals (17). Many challenges contribute to difficulties in applying AW assessment standards, such as diversity of production systems, resource constraints, limited regulations and laws, political and socioeconomic factors, enforcement challenges, and previous assessor training and adaptation (18).

Objective methods for assessing AW that rely on a shorter list of measurements are highly relevant but difficult to identify. Thus, detailed studies that propose and detail new biomarkers to assess AW are needed (15, 19, 20). A promising candidate source for AW indicators is the immune system, which plays a fundamental role in animal health and is intrinsically connected to welfare. Heterophils and lymphocytes are key cells in the immune system and their proportions provide valuable information about the immune status of birds and their acute and chronic status in terms of stress physiology and welfare (21–23). A more detailed consideration of the roles of specific white-blood cell characteristics may contribute to a better understanding of their potential value as AW indicators.

Düpjan and Dawkins (24) have presented evidence suggesting that one pivotal approach to mitigating the risk of diseases and infection lies in effective management practices and improved animal welfare regulation. Additionally, empirical observations indicate that environments fostering well-being over stress and positivity over negativity in both human and animal contexts can reduce susceptibility to disease, potentially resulting in the attenuation of symptoms and faster recovery. On-farm studies are needed to demonstrate that conditions of high welfare degree effectively protect against diseases, whether in experimental conditions or commercial settings. The close relationships among the brain, gut microbiome, immunity, and welfare, alongside established links between mental and physical health, substantiate the significance of high welfare as preventive strategy for disease resistance (24–26).

The induction of distress can elicit alterations in the animal’s immune response, with well-established associations between the hypothalamic–pituitary–adrenal (HPA) axis and immune modulation (27, 28). The white blood cell (WBC) counts and the heterophil to lymphocyte ratio (H/L) have been used as indicators to assess AW, as markers of stress and especially as indicators of chronic distress (29, 30). New developments regarding the analyses of WBC morphology seem relevant in this scenario. During the production cycle, birds can be subjected to chronic stresses, exposure to infectious agents, and other challenging conditions which can result in variations in WBC morphology, with likely increases in toxic change heterophils. Toxic heterophils are leukocytes recognized by a series of morphological characteristics, especially the variability in size, color, and significant changes in granulation. The main characteristics commonly observed include increased cell size, basophilic cytoplasm, with either little or extensive cytoplasmic vacuolization, abnormal granules, hyposegmented nuclei, and nuclear degeneration and degranulation (31, 32).

Considering the importance of searching new AW indicators, first, our background rationale was that the count and the evaluation of the morphology of leukocytes in broiler chickens subjected to intensive farming systems may undergo changes, showing a significant association with the degree of AW. Thus, our guiding hypothesis was that there are associations between both the relative number and the morphological features of broiler chicken leukocytes with scientifically recognized AW indicators at the PH level. This hypothesis served as the foundation of our investigation into the relationship between broiler chicken leukocytes and AW indicators, guiding our analysis and interpretation of the study findings.

The objectives of this study were (1) to study the relative number and morphological features of broiler chicken leukocytes as indicators for AW assessment by verifying their association with scientifically recognized AW indicators at the poultry house (PH) level and (2) to develop a comprehensive cell scoring system, supported by an image guide, focusing on the morphological features of heterophils and lymphocytes, demonstrating the morphological diversity of these cell types, and facilitating easy evaluation and interpretation for assessing broiler chicken welfare. With such objectives, we intended to provide a novel and practical toolkit that enhances our ability to monitor and thus contribute to the improvement in the welfare standards of broiler chickens in farming settings.

## 2 Materials and methods

### 2.1 Description of studied farms and data collection and sample sizes

Field data collection was conducted from 15 November 2022 to 21 November 2022 on farms situated in the municipalities of Dourados, Fátima do Sul, Glória de Dourados, and Laguna Caarapã, 225 km southwest of Campo Grande, the capital of the state. Four intensive poultry production houses, each on a different farm, were visited to assess bird welfare and collect blood samples. Animal procedures were approved by the Animal Use Ethics Committee, Federal University of Parana, protocol 023/2022.

For all PH evaluated, the external temperatures ranged between 25.0 and 35.0°C, indoor relative humidity between 47 and 63%, outdoor between 24 and 76%, indoor air velocity between 3.6 and 1.5 ms-1/h, and the ammonia level between 7.0 and 8.5 ppm, respectively. The light treatments consisted of 18 h of artificial light per day and average illuminance of 13 lx, providing the entire dark period in a single block (from 9 p.m. to 3 a.m.). A questionnaire and flock records were used to obtain general information such as initial number of birds, number of birds at the visit, their breed, age, and mortality and culling rates (Table 1). All visited poultry units were closed houses with black curtains as fixed material to supplement partial walls, climatized with ventilators, exhausters, nebulizers, and automatic drinkers and feeders. The participant farmers raised male Ross birds and operated in an integrated system within the same company. Birds were evaluated when they were 34 to 42 days old (average 5 ± 3 d before slaughter) and weighed on average 2.48 kg. The assessments on each farm were carried out on the same day and conducted in accordance with the procedures and order described in the Welfare Quality (WQ) Assessment Protocol (10). Blood collection followed standard protocols (33).

**Table 1:** Main characteristics of assessed poultry houses (PH), Southwest of Mato Grosso do Sul, Brazil, visited in November 2022.

Variable	PH 1	PH 2	PH 3	PH 4
	Itaporã	Dourados	Glória de Dourados	Laguna Caarapã
Age at visit (days)	34	37	35	42
Flock size during housing (number of birds)	36.250	35.100	34.900	36.350
Flock size during visit (number of birds)	35.283	31.683	33.967	33.697
Mortality (%)	2.67	6.54	1.6	6.18
Culling (%)	1.25	3.2	1.08	1.12
House size, m^2^	2,175	2,400	2,400	2,560
Average weight (kg)	2.50	2.60	2.30	2.53
Feeders	900	1,500	1,000	750
Drinkers	750	750	750	750
Stocking density, birds/m^2^	16.70	14.60	14.50	14.20

During the visit, a partial on-farm welfare assessment was conducted by two assessors using the WQ® protocol for Poultry (10, 34), with the selected measures of lameness, footpad dermatitis, dermatitis on the breast and abdominal areas, hock burn and feather cleaning assessed on ordinal scales, and the Qualitative Behavior Assessment (QBA). The second author was experienced in broiler chicken welfare assessment using the WQ® protocol since 2011 and participated in all the farm visits; the other assessors received adequate training before data collection started. The blood samples were collected after the assessment of all other indicators. The birds were manually restrained for sample collection, which tends to activate stress cascades, even though immobilization was short and gentle. According to current knowledge, acute stress responses are likely not significant for the leukocyte differential count, heterophil/lymphocyte (H/L) ratio, and WBC morphology (32, 35).

The sample size for blood collection was 80 birds per PH, with 320 samples. To determine the sample size, we considered calculations of mean and standard deviation of the H/L ratio in broiler chickens. The formula used for the calculations was n = 2 * SD^2^ * (Zα/2 + Zβ)^2^ / d^2^, where SD is the standard deviation obtained from previous studies or pilot studies, Zα/2 is the critical value corresponding to the desired degree of confidence, Zβ is the power of the statistical test, and d represents the effect size. For this research, we considered a critical value for the degree of confidence of 1.96 (95%) and Zβ of 1.282, as we selected a test power of 90%. To determine the health indicators and the QBA, the sample size was in accordance with that established in the WQ® Assessment Protocol (10).

### 2.2 Blood sample collection

Eighty birds per PH were randomly selected from those not involved in previous WQ® assessments, and approximately 1.0 mL of blood was collected. With a syringe, blood was collected from the wing vein and put into sterilized glass tubes containing ethylene diamine tetra-acetic acid (EDTA) to prevent blood clotting.

### 2.3 Assessment of environmental indicators

Environmental parameters were measured to describe the indoor living conditions in all units, with the assessment of health indicators. All data were obtained at bird level, from equidistant locations. Temperature and relative humidity were assessed with Akso AZ77535 (Honk Kong, China). To measure air velocity, ammonia concentration (NH3), and light intensity, we used the following meters: digital thermo-hygrometer-decibelimeter-luximeter-anemometer (THDLA—500) and single NH3 detector, respectively. The results for both health indicators and environmental parameters are shown in Table 2.

**Table 2:** Temperature, air velocity, light intensity, relative humidity, and ammonia (NH3) assessed poultry houses, Southwest of Mato Grosso do Sul, Brazil, visited in November 2022.

Variable	PH 1	PH 2	PH 3	PH 4
	Itaporã	Dourados	Glória de Dourados	Laguna Caarapã
Outdoor temperature (°C)	32.00	30.20	29.50	25.40
Indoor temperature (°C)	31.10	28.50	27.00	26.25
Indoor air velocity (m/s)	2.20	3.20	2.50	2.00
Outdoor air velocity (m/s)	1.60	3.10	3.60	1.50
Light intensity (lx) indoor	15	15	12	10
Light intensity (lx) outdoor	>20.000	>20.000	>20.000	12,810
Indoor relative humidity (%)	47.00	55.20	51.00	63.50
Outdoor relative humidity (%)	24.70	47.20	45.00	76.00
Ammonia (ppm)	7.50	9.00	8.00	7.00

### 2.4 Bird affective states

The QBA was performed in all houses. This qualitative analysis is a methodology that considers the body language of the birds, i.e., the expressive quality of how birds behave and interact with each other and the environment. The flocks were assessed as described in the WQ® protocol (10), and 25 behavioral expressions were scored on visual analogue scale (VAS). The absence of behavioral expression was coded in blue and the maximum expression was coded in red (6, 10, 34, 36). The behavioral terms developed for Brazilian Portuguese native speakers (37) used were attentive, lethargic, apathetic, bored, agitated, frustrated, with pain, uneasy, disturbed, scared, fearful, distressed, desperate, relaxed, active, interested, confident, calm, peaceful, inquisitive, playful, vitality, aggressive, comfortable, and positively occupied.

### 2.5 Health assessment

**Figure 1:**
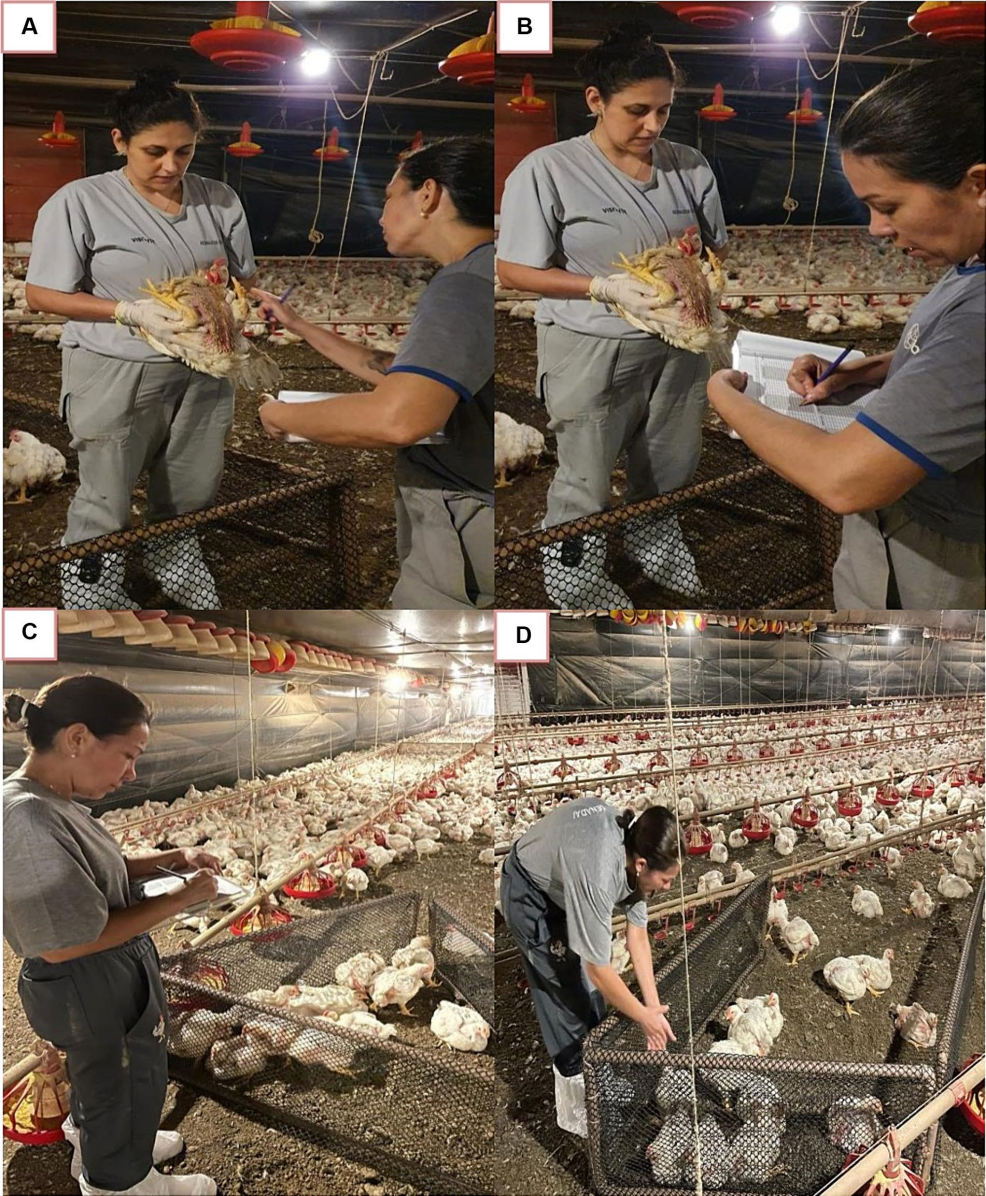
Evaluation bird health indicators **(A–D)** using the Welfare Quality^®^ Protocol of poultry houses in the Southwest of Mato Grosso do Sul, West Center of Brazil.

Health assessments were performed in all PH, according to procedures described in the WQ® protocol. Mobile plastic enclosures were used to separate the birds selected for evaluation (Figures 1A–D). In total, 10 birds in 10 locations of the PH were randomly selected for the assessment of 100 birds per PH. Birds were manually held for the assessment of breast dermatitis and abdominal lesion (4-score scale, where zero was absence of lesion and three severe lesion), plumage cleanliness (4-point scale, where zero identified clean and three very dirty plumage), pododermatitis (5-point scale, where zero was lesion absence and four severe lesion), and hock burn (3-point scale, where zero was lesion absence and two severe lesion). All health indicators were assessed on the same sample of 100 birds per farm by the same assessor (10, 34). The last health indicator assessed was lameness, where other 150 birds per farm were individually stimulated to walk, and a visual inspection of walking ability was conducted and scored with a six-point scale, where zero was normal gait and five was given for birds that were unable to walk. The selected location was based on the WQ® protocol applied to broiler chicken. The location in the PH was generated by computer. All the PH were in the same size (10).

In view of the varied nature of the scoring systems and in order to better demonstrate and interpret the results, we classified the scores as follows: score 0 (normal); score 1 (moderate abnormality); and scores 2 to 3 (severe abnormality) for breast dermatitis and abdominal lesion and plumage cleanliness; score 0 (normal); score 1 (moderate abnormality); score 2 to 4 (severe abnormality) for pododermatitis and hock burn; and for lameness, the classification was score 0–1 (normal); scores 2 to 3 (moderate abnormality); and scores 4 to 5 (severe abnormality).

### 2.6 Blood analysis

The blood samples were processed, and a blood smear was prepared immediately after collection. For each bird, one drop of blood was put onto a glass slide, and with the use of an extender, the smear was performed. After being air-dried, smears were fixed and stained using a rapid hematology stain (PA205, Newprov, Brazil) or Wright’s Giemsa solution (PA202, Newprov, Brazil). Samples were processed in the hematology laboratory at Unigran and Diagnostico lab, which were located at Dourados and Campo Grande, MS.

### 2.7 Differential counts and cell scoring system

The differential WBC counts were performed using an optical microscope (MOC), Carl Zeiss Microscopy GmbH, Axio Scope A1 model, with the assistance of ZEN Lite (Blue Edition) imaging software, at magnifications of 1,000×. One hundred leukocytes, including heterophil, young heterophil, lymphocyte, eosinophil, and monocyte cells, were counted in at least 10 fields per slide. The images of the smears were photographed using an Axiocam 503 color camera connected to the MOC. We adopted the morphological criteria for heterophil sorting as described by (32, 38). Abnormal heterophil and lymphocytes were included in the differential counts. A cell score was created for recognition, classification, and interpretation of morphologic diversity of broiler chicken heterophils and lymphocytes. The heterophil scores were from zero (absence of change in cell morphology) to four (severe change in cell morphology), and the lymphocyte scores were from zero (absence of change in cell morphology) to one (with change in cell morphology). We further classified the scores as score 0 (normal), scores 1 to 2 (moderate abnormality), and scores 3 to 4 (severe abnormality) (Table 3). The counts and the analyses of morphological characteristics of WBCs were conducted, and qualitative interobserver reliability verification was applied based on simple comparison of 48 independent blood sample analyses by two assessors, i.e., 12 readings per PH or 15% of all blood samples analyzed.

**Table 3:** Description of the morphologic diversity observed on the blood films of bird heterophil and lymphocyte cells for their classification, as per morphological criteria described by Stacy et al. (32) and Clark et al. (38).

Cell	Description of the morphologic	Classification	Images
Heterophil normal (0)	Normal morphology; Cytoplasm filled with a large number of fusiform granules (red) and with slight basophilia. Bi- or tri-lobed nucleus with dense chromatin (dark blue).	C0	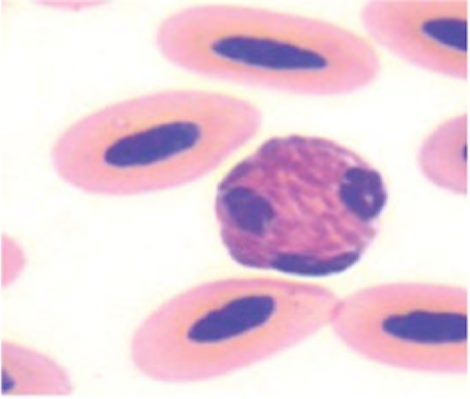
Heterophil abnormal (1)	Light alteration (reversible lesion); cytoplasmic granules less fusiform and reddish in color. Bi- or tri-lobed nucleus with dense chromatin (dark blue).	C1	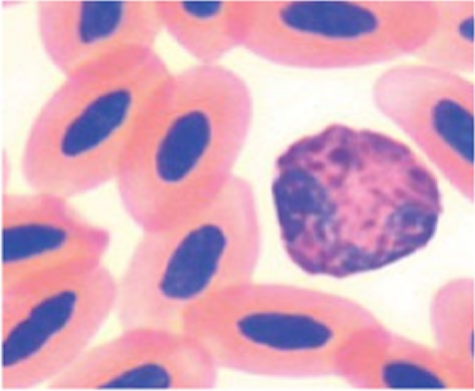
Heterophil abnormal (2)	Moderate alteration (reversible lesion); cytoplasmic granules large, round and in smaller quantities. Nucleus with reduced segmentation, with less dense chromatin (light blue).	C2	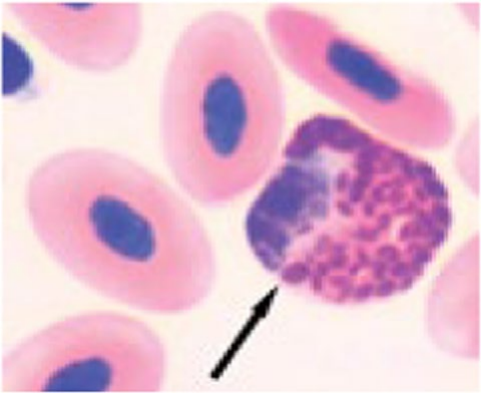
Heterophil abnormal (3)	Severe alteration (reversible lesion); cytoplasmic granules large, round, fewer and with vacuoles present. Nucleus with reduced segmentation, with less dense chromatin (light blue).	C3	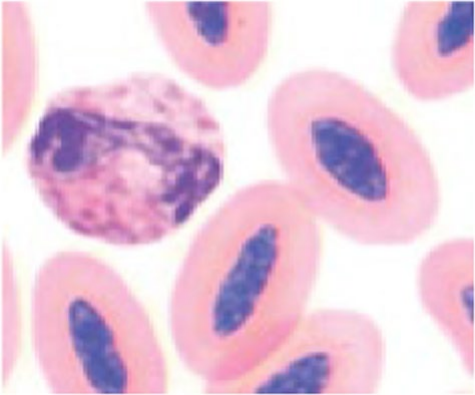
Heterophil abnormal (4)	Severe alteration (irreversible damage); heterophils exhibit marked morphological atypia: cytoplasm with larger, round granules, but with few or no granules, presence of vacuoles and intense basophilia. Nucleus with loose chromatin. Presence of young cells (band, metamyelocyte and myelocyte).	C3	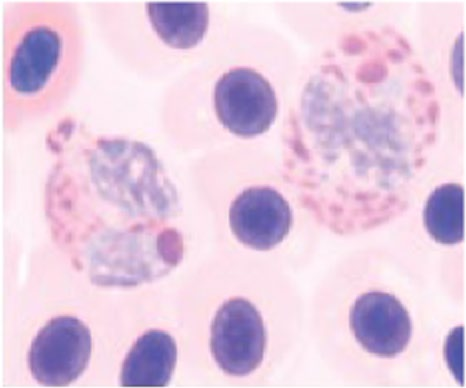
Lymphocyte normal (0)	Normal morphology; basophilic cytoplasm; circular nucleus with dense chromatin (dark blue), occupying 70 to 90% of the cytoplasm.	C0	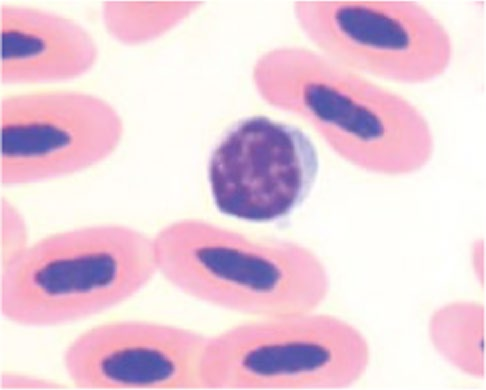
Lymphocyte abnormal (1)	Abnormal morphology; Increased cytoplasm with intense basophilia	C1	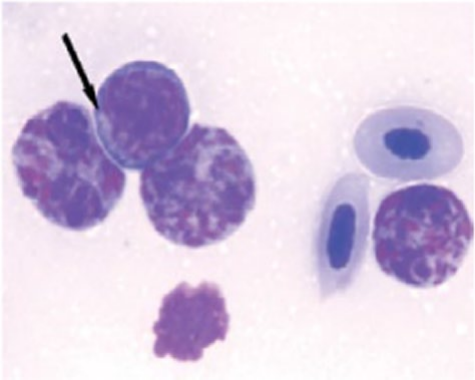

### 2.8 Determination of heterophil to lymphocyte (H/L) ratio

The H/L ratio was calculated for all birds from whom blood was collected. The H/L ratio was calculated by dividing the number of heterophils and the number of lymphocytes (39).

### 2.9 Statistical analysis

Descriptive statistics were used to verify the main characteristics observed per PH as follows: environmental indicators (temperature, relative humidity, air velocity, NH3 and light intensity); bird affective states (attentive, lethargic, apathetic, bored, agitated, frustrated, with pain, uneasy, disturbed, scared, fearful, distressed, desperate, relaxed, active, interested, confident, calm, peaceful, inquisitive, playful, vitality, aggressive, comfortable, and positively occupied); health indicators (breast dermatitis and abdominal lesion, plumage cleanliness, pododermatitis, and lameness); differential count leukocytes; and classification and interpretation of morphologic diversity cell.

The differences between the proportions of heterophils, lymphocytes, monocytes, and heterophil to lymphocyte ratio for the birds in the different PH were transformed to a binomial outcome by considering the number of heterophils, lymphocytes, and monocytes observed in 100 blood cells. The binomial generalized linear model (GLM) is the usual regression approach for this type of data. A Quasi-likelihood approach was used to prevent misspecification problems. Residual analysis was carried out to assess the model fitting. Such information is available along the statistical analysis section and now presented in Supplementary Table S5.

For the health variables, breast and abdominal dermatitis, plumage cleanliness, pododermatitis, hock burn and lameness, and regression models for ordinal responses were fitted to evaluate the association between the PH and bird health conditions. In this case, the proportional odds regression model was used to properly evaluate and compare the PH. The Wald and likelihood ratio tests (LRT) were considered to conclude about the differences between the PH under the quasi-binomial and proportional odds regression models, respectively. The results were considered statistically significant when *p* < 0.05. For both blood and health indicators, when the Wald or LRT tests pointed out a significant result, we carried out Tukey’s honestly significant differences (Tukeys’ HSD) procedure to compare the pairs of PH ensuring a 5% global significance level.

For the QBA individual terms, a heat map was elaborated for a better visualization of more prevalent adjectives. This is a method used in unsupervised machine learning to group similar data points into clusters. The algorithm organizes the data in a hierarchical structure, where clusters of data points are grouped together based on their similarities.

The PH was considered as a fixed rather than a random effect due to the small number and high variability of available PH. We opted to compare the results of the four available PH, rather than extrapolating these results for a more general population of PH.

Finally, all analyses were carried out using R software for statistical computation version 4.3.1 (40). The R libraries geepack, ordinal, and emmeans were used for the regression analysis (41–43).

## 3 Results

### 3.1 Bird affective states

**Figure 2:**
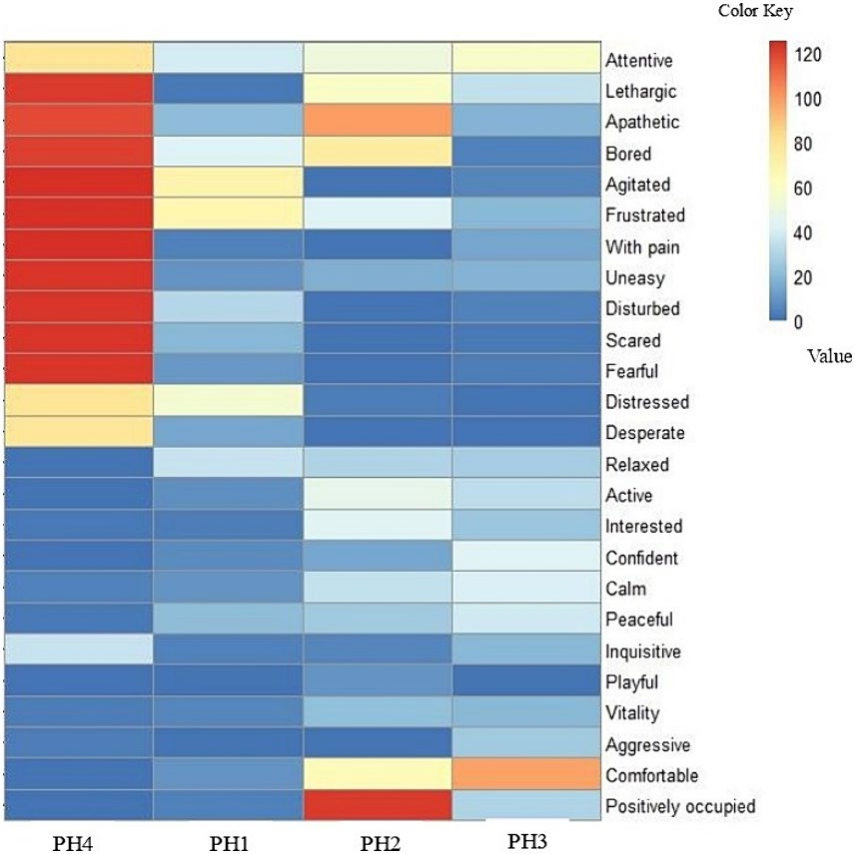
A heatmap of bird affective states in poultry houses (PH), located in the Southwest of Mato Grosso do Sul, Brazil. The assessment of the flocks was conducted following the WQ protocol (10). The color bar on the right side represents the visual analogue scales (VAS) for the 25 behavioral expressions that were scored. The absence of behavioral expression is indicated in blue, and the maximum expression in red.

In the assessment and classification of the QBA, two groups of behavior were identified (group 1—“Attentive” to “Desperate” and group 2—“Relaxed” to “Positively occupied”). A heat map (Figure 2) was generated based on the scoring of the 25 behavioral expressions observed in the birds from the data set obtained by the QBA. The color bar on the right side demonstrates the VAS.

The absence of a specific behavioral expression is indicated in blue, and its maximum expression is indicated in red (Figure 2). The PH 4 differed from the other farms in that it had higher frequency of behaviors of the group 1: attentive, lethargic, apathetic, bored, agitated, frustrated, with pain, uneasy, disturbed, scared, fearful, distressed, and desperate. These behaviors in the map are determined by redder areas. In contrast, poultry houses 1, 2, and 3 had lower frequency of group 1 behaviors and were classified in group 2: relaxed, active, interested, confident, calm, peaceful, inquisitive, playful, vitality, aggressive, comfortable, and positively occupied. These behaviors in the map are represented by blue color and its variations.

### 3.2 Health assessment

**Figure 3:**
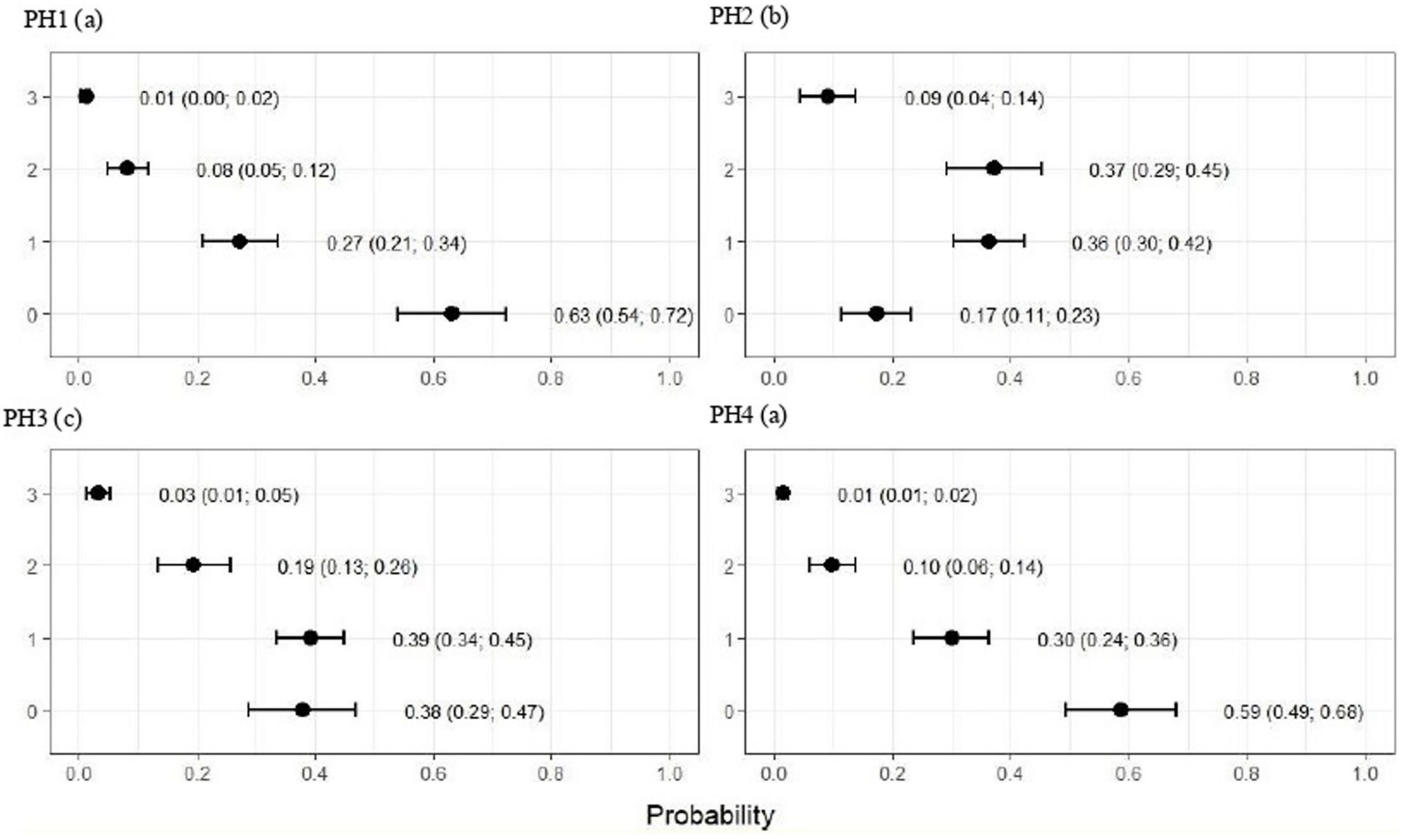
Estimated score probabilities of breast and abdominal dermatitis provided by the proportional odds regression model in birds from four poultry houses (PH) in the Southwest of Mato Grosso do Sul, Brazil; different letters (a), (b) and (c) after house number indicate differences among barns (*p* < 0.05).

**Figure 4:**
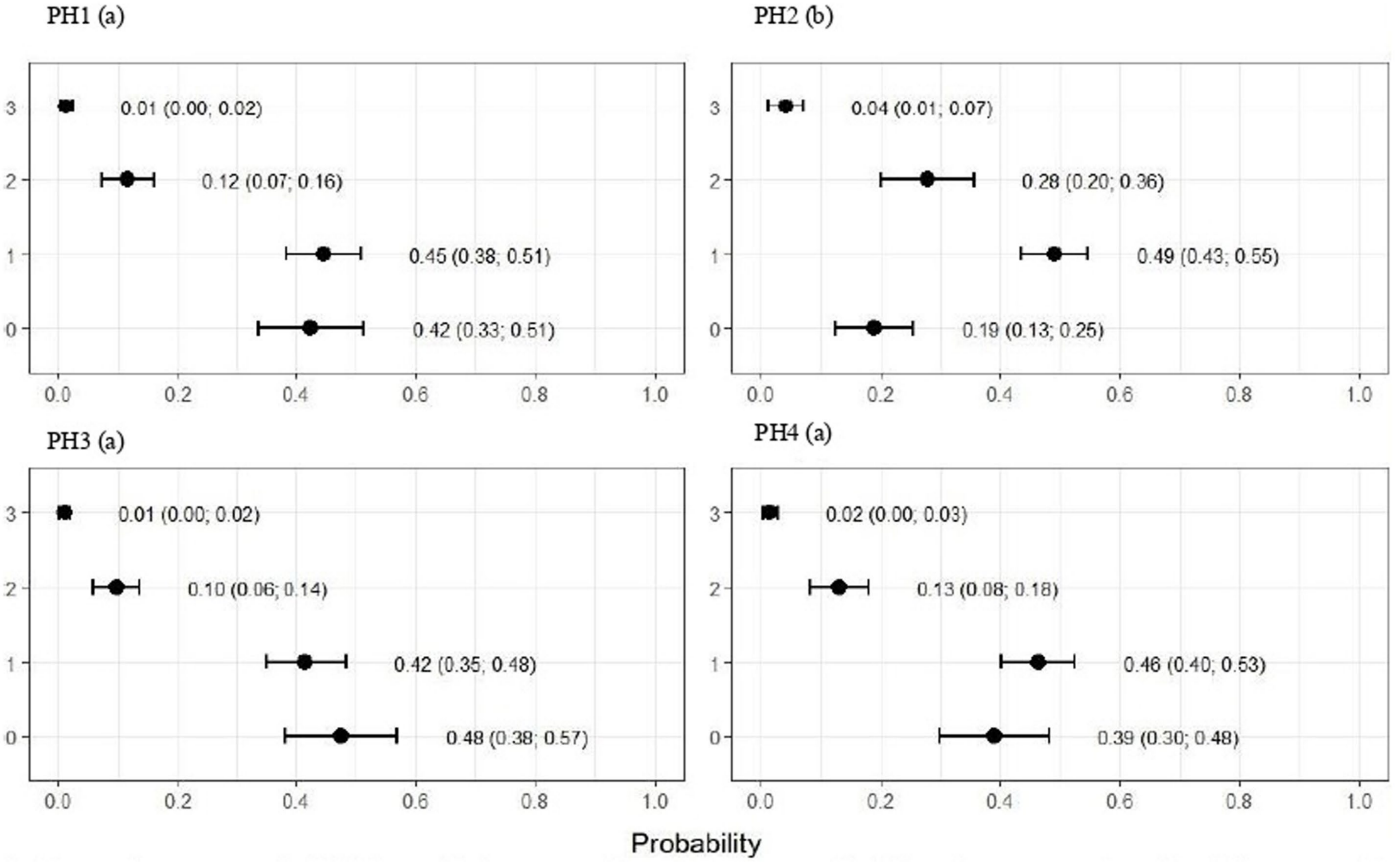
Estimated score probabilities of plumage cleanliness provided by the proportional odds regression model in birds from four poultry houses (PH) in the Southwest of Mato Grosso do Sul, Brazil; different letters (a), (b) and (c) after house number indicate differences among barns (*p* < 0.05).

**Figure 5:**
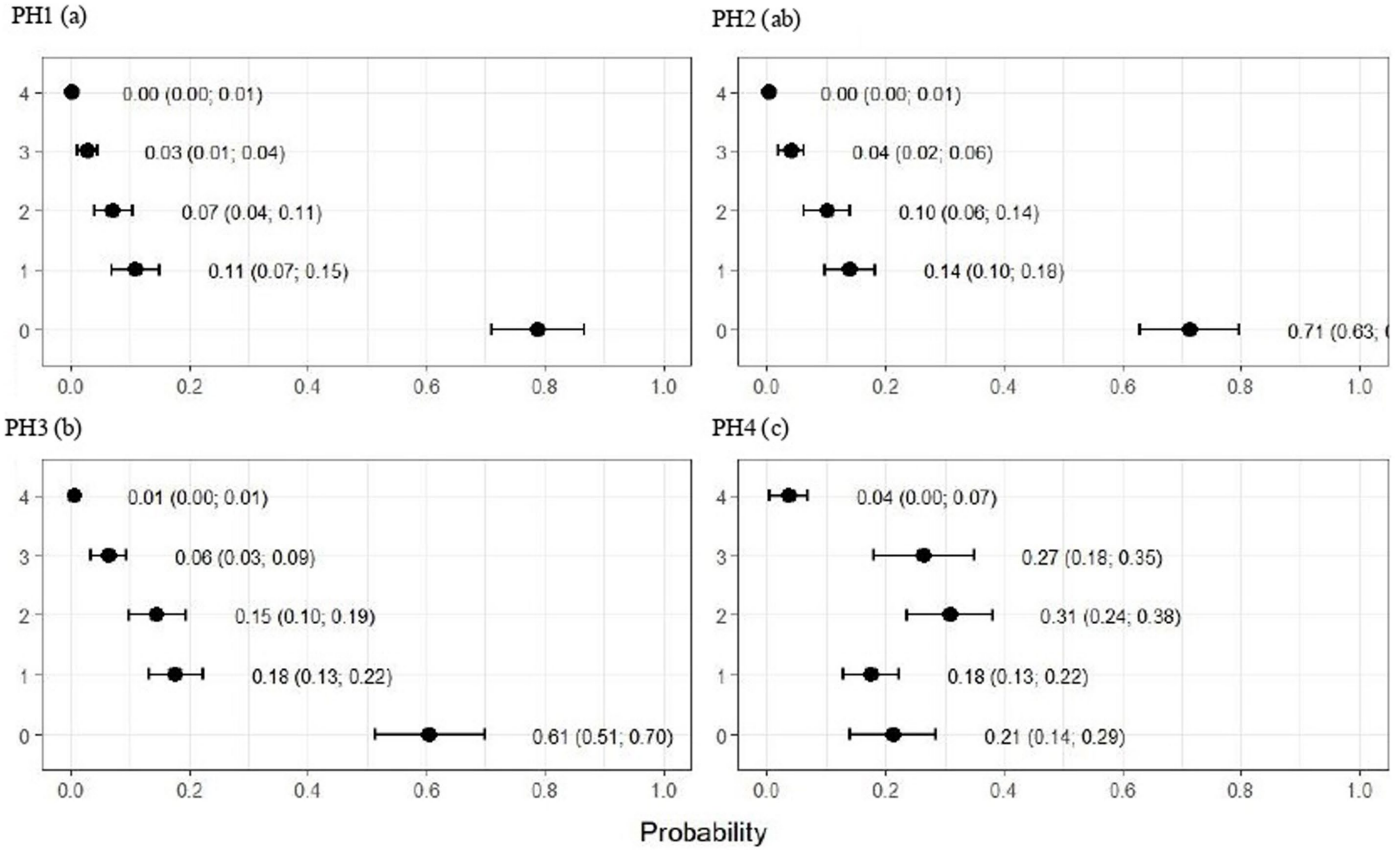
Estimated score probabilities of pododermatitis provided by the proportional odds regression model in birds from four poultry houses (PH) in the Southwest of Mato Grosso do Sul, Brazil; different letters (a), (b) and (c) after house number indicate differences among barns (*p* < 0.05).

**Figure 6:**
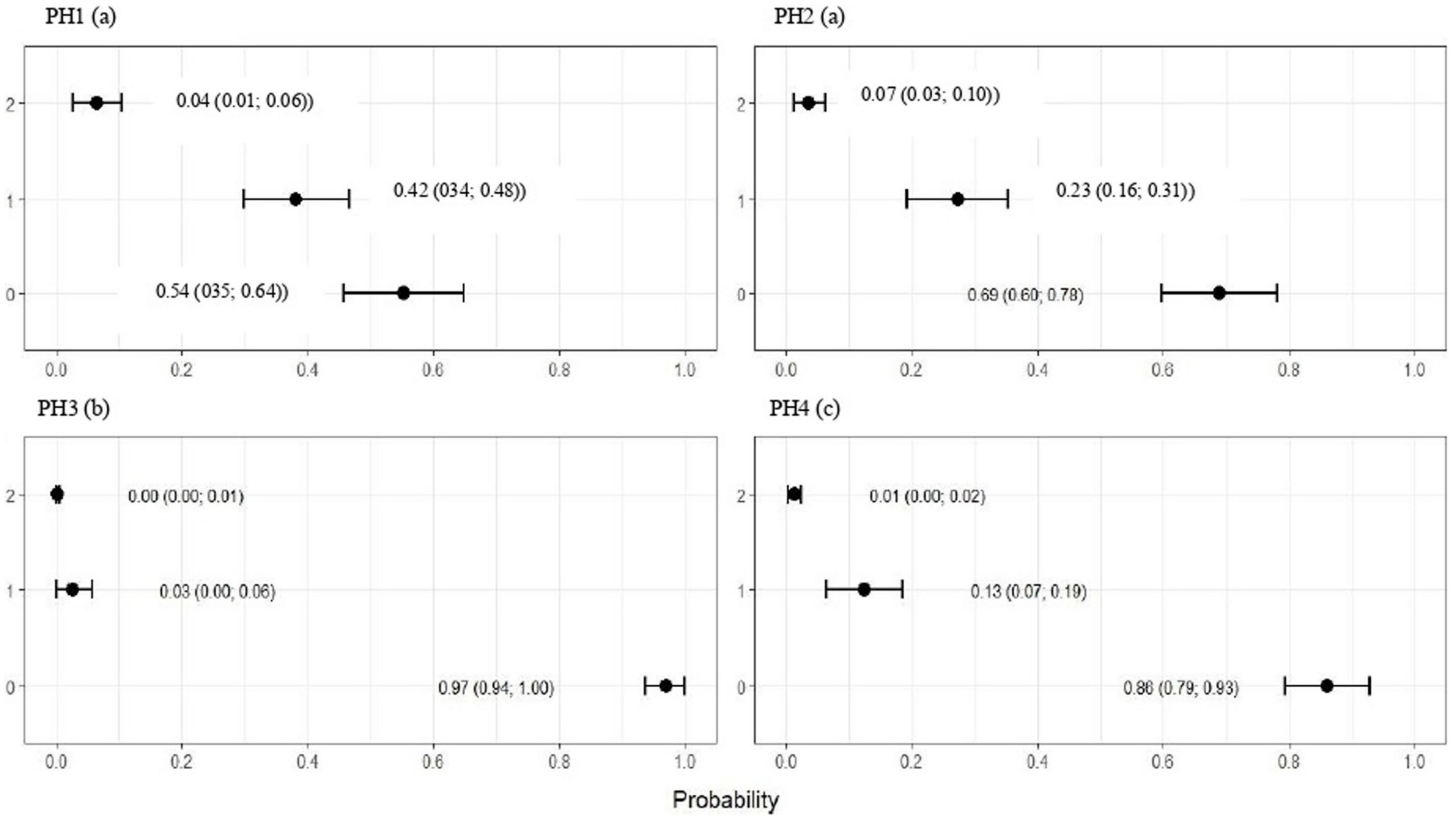
Estimated score probabilities of hock burn provided by the proportional odds regression model in birds from four poultry houses (PH) in the Southwest of Mato Grosso do Sul, Brazil; different letters (a), (b) and (c) after house number indicate significant differences among barns (*p* < 0.05).

**Figure 7:**
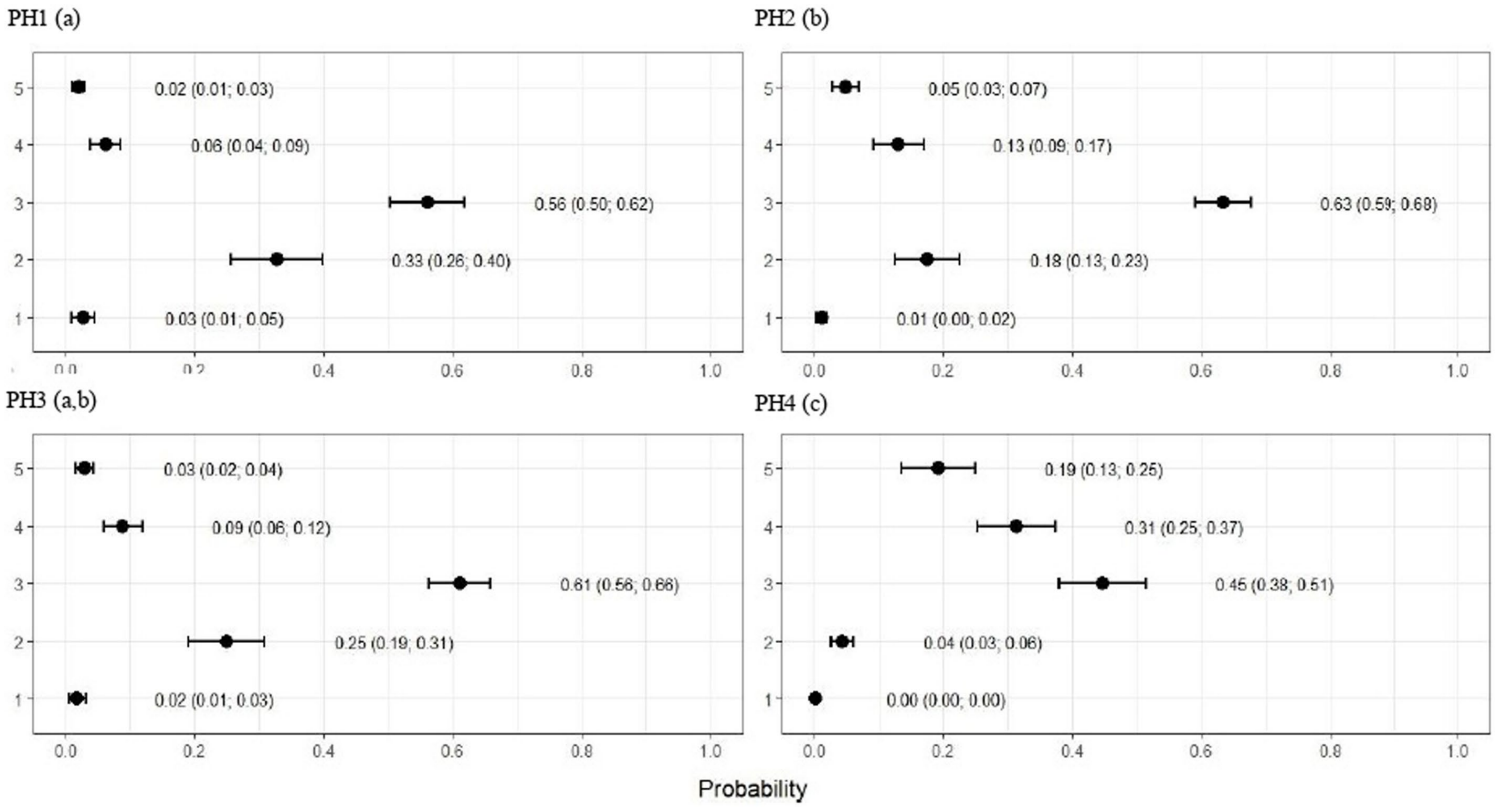
Estimated score probabilities of lameness provided by the proportional odds regression model in birds from four poultry houses (PH) in the Southwest of Mato Grosso do Sul, Brazil.; different letters (a), (b) and (c) after house number indicate significant differences among barns (*p* < 0.05).

For all health indicators, there were differences among houses (*p* < 0.001) (Figures 3–7). Figure 3 shows a higher frequency of problems observed for PH 2, in which the proportions of birds with scores 2 (moderate) and 3 (severe) were estimated at 0.37 (CI95%: 0.29; 0.45) and 0.09 (CI95%: 0.04; 0.14), respectively (*p* < 0.05).

For PH 3, the results were intermediate, where the proportions of birds with score 2 (moderate) were estimated at 0.19 (CI95%: 0.13; 0.26) and with score 3 (severe) at 0.03 (CI95%: 0.01; 0.05) with *p* < 0.05. Moreover, PH1 and 4 observed a lowest frequencies of breast and abdominal dermatitis, where the proportions of birds with score 2 (moderate) were estimated at 0.08 (CI95%: 0.05; 0.12) and 0.10 (CI95%: 0.06; 0.14) and with score 3 (severe) at 0.01 (CI95%: 0.00; 0.02) and 0.01 (CI95%: 0.01; 0.02), respectively, and *p* < 0.05 (Figure 3).

The plumage scores for PH2 showed that the proportions of birds with scores of 1 (light) and 2 (moderate) were estimated at 0.49 (CI95%: 0.43; 0.55) and 0.28 (CI95%: 0.20; 0.36), respectively (*p* < 0.05). For birds from the other houses, there was no significant difference in plumage cleanliness, as shown in Figure 4. As Figure 5 showed, pododermatitis lesions were more frequent in PH4. The proportions of scores equal to 2 and 3 were estimated at 0.31 (CI95%: 0.24; 0.38) and 0.27 (CI95%: 0.18; 0.35) and *p* < 0.05, respectively. For hock burn, the scores were worse in birds from PH 1, where the proportions of birds with scores of 1 (moderate) and 2 (severe) were estimated at 0.42 (CI95%: 0.34; 0.48) and 0.04 (CI95%: 0.01; 0.06), respectively. For PH 2, the results were intermediate to score 1 (0.23, CI95%: 0.13; 0.31). The proportions of birds in PH 3 and 4 with a score of 1 (moderate) were estimated at 0.03 (CI95%: 0.00; 0.06) and 0.13 (CI95%: 0.07; 0.19) and PH 2 (severe) were estimated at 0.00 (CI95%: 0.00; 0.01) and 0.01 (CI95%: 0.00; 0.02), with *p* < 0.05, respectively. All results are shown in Figure 6.

As observed in Figure 7, the birds from PH 4 were most affected by lameness, where the proportions of birds with scores of 4 (severe abnormality) and 5 (incapable of walking) were estimated at 0.31 (CI95%: 0.25; 0.37; *p* < 0.05) and 0.19 (CI95%: 0.13; 0.25; *p* < 0.05.), respectively. For the other houses, the scores were lower in birds from PH 1 compared with PH 2 and 3, where intermediate results were observed.

### 3.3 Differential counts and cell morphology

The results obtained revealed that the relative heterophil count was higher compared with the reference values, indicating relative heterophilia in two PH. According to Thrall et al. (33), in addition to being significantly different among PH (*p* < 0.05), the parameters analyzed corresponded to the upper ranges of 15 to 50% of reference values reported for healthy birds.

**Figure 8:**
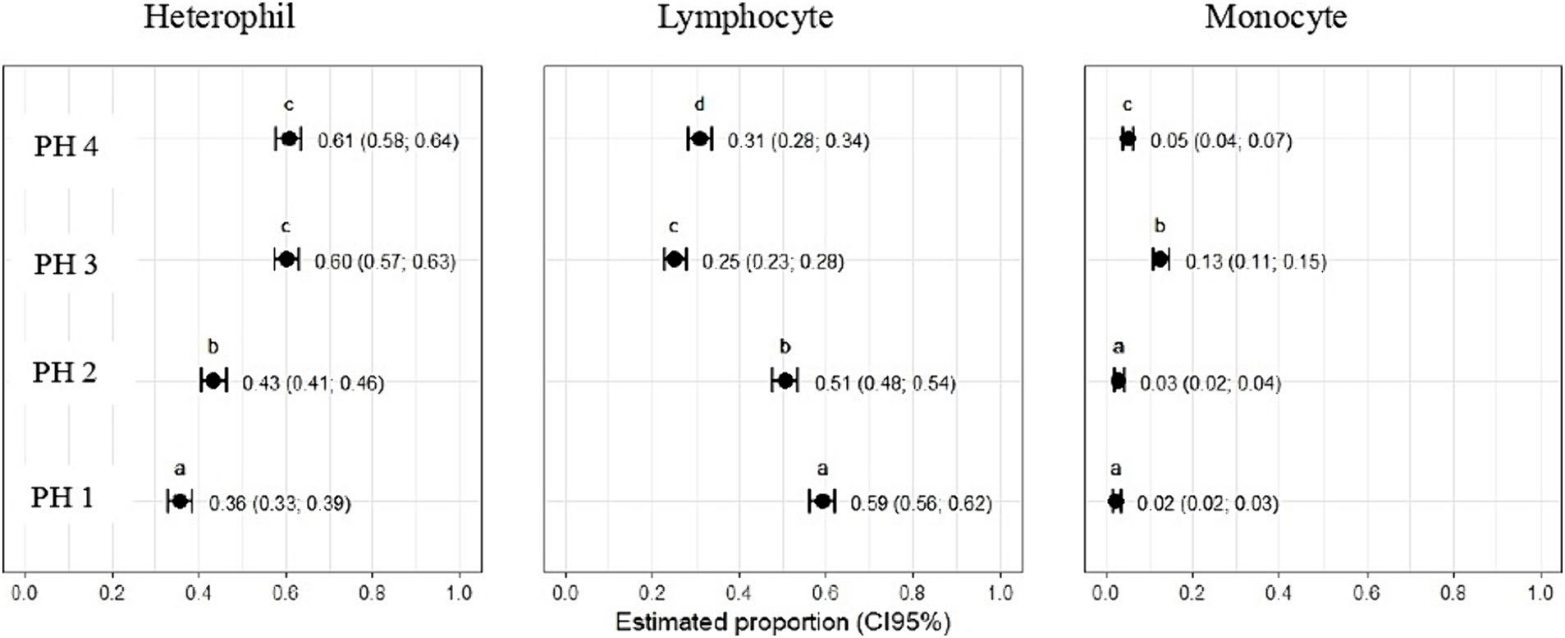
Hematological parameters of birds in four poultry houses (PH) in the Southwest of Mato Grosso do Sul, Brazil; different letters (a), (b) and (c) indicate significant differences among barns.

Statistical differences revealed that the estimated proportion of heterophils was higher in birds from PH4 (*p* = 0.61, CI95%: 0.58; 0.64) and birds from PH3 (*p* = 0.60, CI95%: 0.57; 0.63), with p < 0.05, respectively (Figure 8). In PH2 birds, the results were intermediate (*p* = 0.43, CI95%: 0.41; 0.46; *p* < 0.05), while for PH1, a lower result was observed for the heterophil count (*p* = 0.36, CI95%: 0.33; 0.39). For lymphocytes, the estimated proportion in the blood of birds in the PH1 group was 0.59 (*p* = 0.59), with a 95% confidence interval (CI 95%) ranging from 0.56 to 0.62 with *p* < 0.05. For birds in PH2, the proportion of lymphocytes was 0.51 (*p* = 0.51), with a CI 95% ranging from 0.48 to 0.54 (*p* < 0.05); in PH4, the estimated proportion was 0.31 (*p* = 0.31), with a CI 95% ranging from 0.28 to 0.34, and finally, for birds in PH3, the estimated proportion of lymphocytes was 0.25 (*p* = 0.25), with a CI 95% ranging from 0.23 to 0.28. Based on these results, birds from PH1 had the highest proportion of lymphocytes, followed by PH2, PH4, and PH3 in descending order.

The estimated proportion of monocytes was higher for PH3 birds 0.13 (*p* = 0.13, CI95%: 0.11; 0.15), followed by PH4 birds 0.05 (*p* = 0.05, CI95%: 0.04; 0.07), PH2 birds 0.03 (*p* = 0.03, CI95%: 0.02; 0.04), and PH1 birds 0.02 (*p* = 0.02, CI95%: 0.02; 0.03). There was no significant difference among the proportion of monocytes in the blood of birds from PH2 and PH1 (Figure 8).

**Figure 9:**
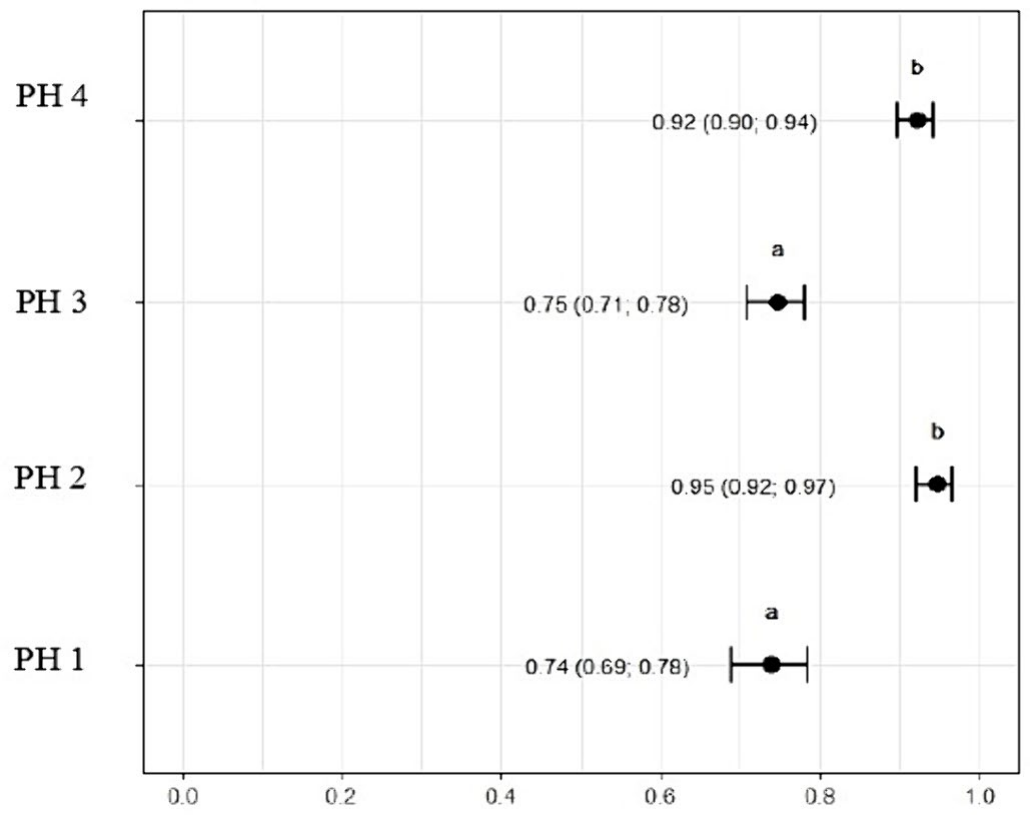
Heterophils (score 3 and 4) count and morphology in blood samples of birds from four poultry houses (PH) in the Southwest of Mato Grosso do Sul, Brazil; different letters (a), (b) and (c) indicate significant differences among PH.

**Figure 10:**
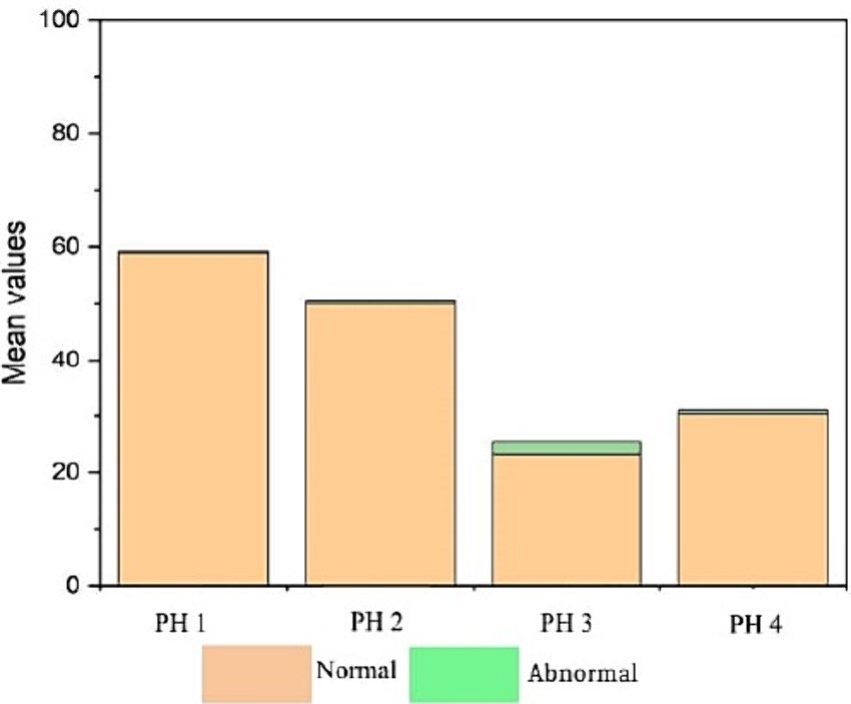
Lymphocyte count and morphology in blood samples of birds from four poultry houses (PH) in the Southwest of Mato Grosso do Sul, Brazil.

**Figure 11:**
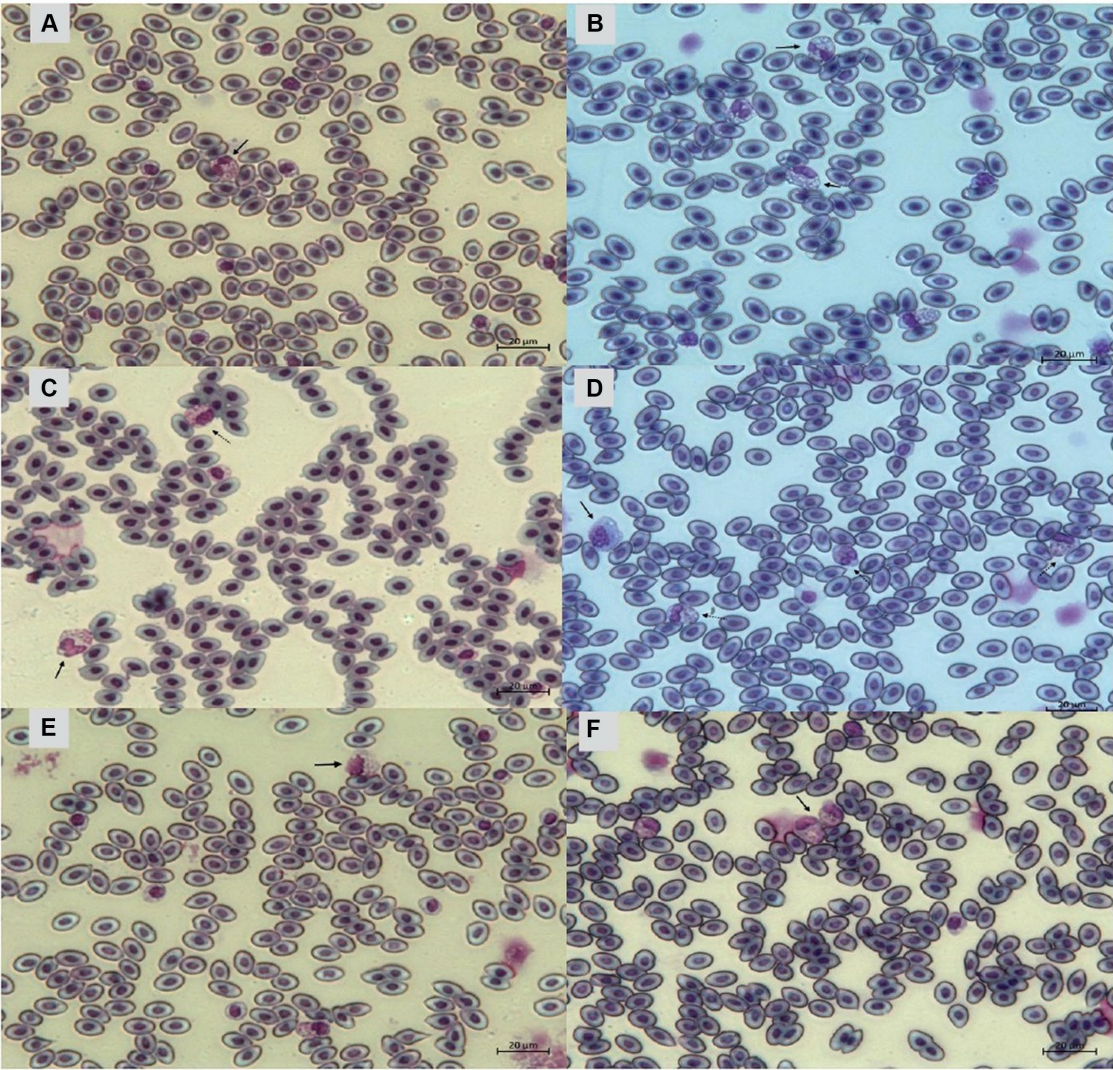
Heterophils of birds (*Gallus gallus* domesticus) in four poultry houses (PH) in the Southwest of Mato Grosso do Sul, Brazil. Cell morphology were evaluated according to Table 3 for comparative purposes. Images **(A,B)** represents heterophil of the classification 3, severe alteration (reversible lesion). Images **(C–F)** represents heterophils abnormal of the classification 4, severe alteration (irreversible lesion). Methanolic Wright-Giemsa stain. The hematological slides were analyzed using an Optical Microscope (MOC), Carl Zeiss Microscopy GmbH, Axio Scope Al model, with the assistance of ZEN Lite (Blue Edition) imaging software, at magnifications of 1,000×. The images of the smears were photographed using an Axiocam 503 color camera connected to the MOC.

Figure 9 indicates that birds from PH2 and PH4 showed more critical results in terms of heterophil morphology, i.e., they had a higher proportion of heterophils with more than 90% of cells with scores of 3 and 4, which was classified as toxic change heterophils due to severe abnormality, while birds from PH1 and PH3 had a proportion of approximately 70% of cells with scores of 3 and 4 (*p* < 0.05). The birds from all houses presented normal morphological characteristics for the most lymphocytes (score 0) and exhibited abnormal form only in 0.9 ± 0.1% (score 1), with one to at most six abnormal cells per smear (Figure 10). Figure 11 shows the images of the heterophil morphology of birds (*Gallus gallus domesticus*) in our study.

### 3.4 Heterophil to lymphocyte ratio (H/L)

The ratio of heterophils to lymphocytes was higher for birds in PH3 3.03 (*p* = 3.03, CI95%: 2.65; 3.42) and PH4 2.58 (*p* = 2.58, CI95%: 2.19; 2.98). For PH2 birds, the results were intermediate 1.03 (*p* = 1.03, CI95%: 0.90; 1.15), while for PH1 birds, the proportion was lower 0.70 (*p* = 0.70, CI95%: 0.60; 0.79) (Figure 11).

## 4 Discussion

Our results provide insights into the potential contribution of qualitative leukocyte assessment, as it seems to provide relevant information for broiler welfare assessment. Despite the universally central role of heterophils in inflammatory and infectious responses in vertebrate species, the literature on the use of cell morphology as a diagnostic tool for welfare remains limited.

The analysis of counts and morphological characteristics of WBC and the on-field welfare assessments for birds in the same PH presented an interesting range of results which suggest the potential of heterophil morphological abnormalities as a contribution to bird welfare assessment. Even though the four PHs were classified as intensive confinement systems, which are generally characterized by low welfare conditions (6, 37, 44), there was important welfare variation among them. These results allow the investigation of how welfare assessments carried out in the field related to physiological indicators obtained from blood samples, thus contributing to a better understanding of poultry welfare in intensive confinement systems. The welfare of broiler chickens is significantly influenced by internal environmental conditions within PH, including temperature, humidity, litter quality, ventilation, illuminance, and gas concentrations such as NH3 and carbon dioxide (CO2) (45–47). Our results showed that birds had different welfare levels, although the internal environmental conditions were within the regulatory limits for intensive chicken production systems. Coelho et al. (48) highlighted the importance of careful attention to the environmental conditions in intensive poultry production systems, especially in tropical climates, because external climate interacts differently with internal environmental conditions. The variation allowed for the investigation of how welfare assessment conducted on field related to immunological indicators obtained from blood samples.

Hematology may contribute to the monitor AW and health of farm animals, as it tends to reflect the impacts animals undergo concerning their welfare, including chronic impacts. More specifically, leukocytes play a vital role in the immune response and are involved in various inflammatory processes (25, 49–51). There is a tradition for considering WCB count and H/L ratio to contribute to AW assessment, and our results confirm such contribution. Notably, the percentage of heterophils was higher on PH 4 and 3, suggesting potential stress responses in the birds on these farms. Heterophils have a fundamental role in the stress response and acute inflammation in birds (23, 52). Our original results in terms of the significant differences in the morphology of heterophils comparing birds from different PH suggest diagnostic power for this analysis as well, especially considering that differences seemed consistent with observed for on-field welfare assessment.

The percentages of lymphocytes also differed, and the PH 1 presented the highest counts. Such differences in blood profiles suggest variations in the birds’ immunological and physiological responses, which may likely be attributed to different factors related to living conditions and environmental stimuli (30, 53), habitat alterations (54), hygienic status of feed, water and environment, pathogen exposure (55), the gut (56), and the use of antibiotics and chemotherapeutics (57). These are all factors which warrant further research.

The H/L ratio is a crucial stress indicator in poultry (58). The study revealed that PH3 had the highest H/L ratio, followed by PH 4 and 2. This suggests that the birds in these poultry farms may be experiencing more stress in comparison to birds on poultry farm 1, which had the lowest H/L ratio. The PH 2, despite showing intermediate results for cell proportions, remained with an H/L value above the reference value, revealing poor welfare conditions and high levels of stress (59). These findings align with the assessment of health indicators and blood variables as percentage and morphology of heterophil, collectively indicating variations in stress levels among the PHs, as reported in the literature (14, 23, 39, 58, 60).

**Figure 12:**
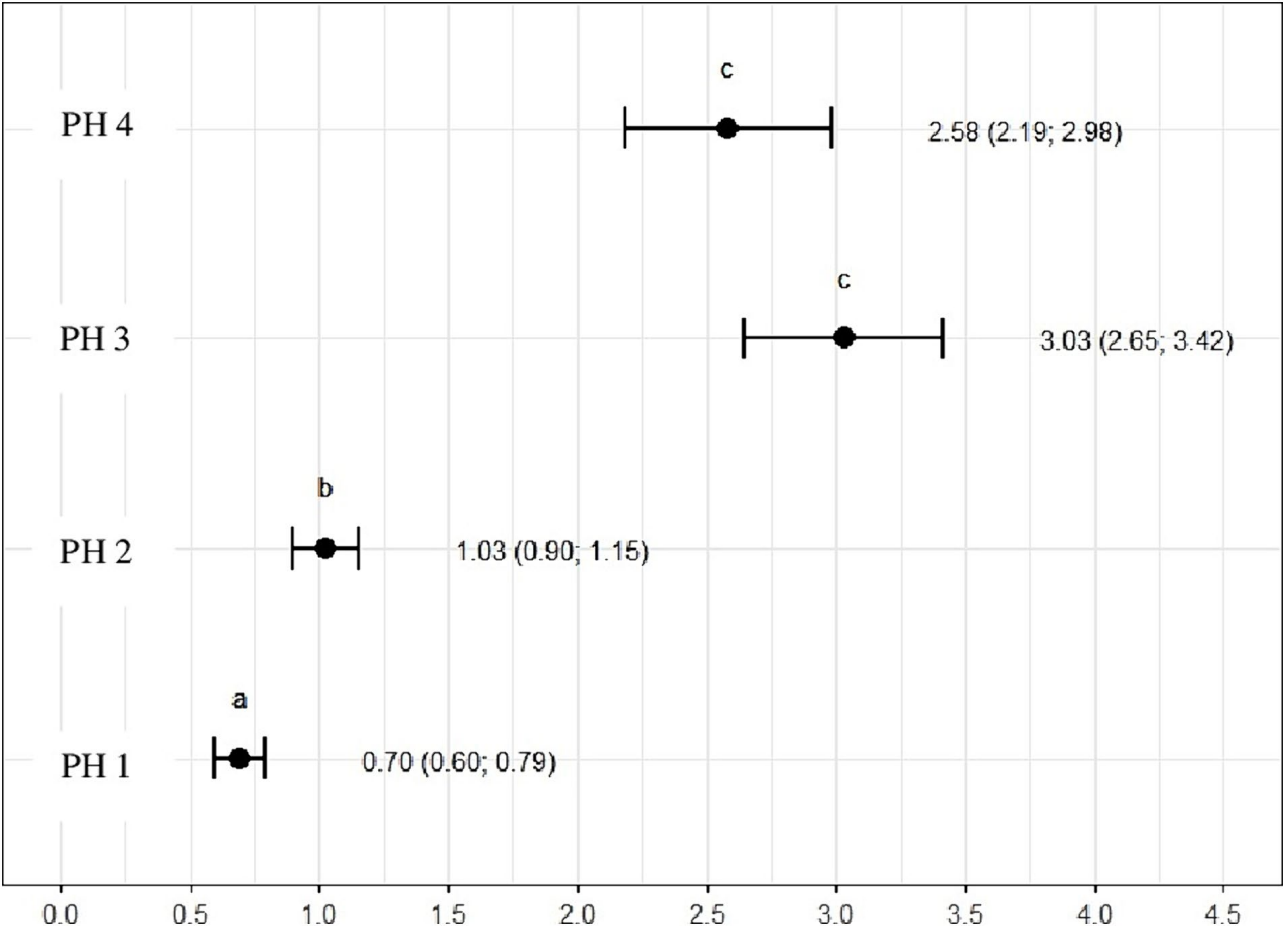
Heterophil and lymphocyte ratio (H/L) in the blood samples of birds from four poultry houses (PH) in the Southwest of Mato Grosso do Sul, Brazil; different letters (a), (b) and (c) indicate significant differences among barns.

Birds from all PH presented high scores of toxic change heterophils (Figure 12), with those from PH2 and PH4 showing the worst results. Stacy et al. (32) described that heterophils and neutrophils exhibited morphological abnormalities under significant inflammatory stimulus, and these changes were the result of accelerated production demanded by the organism, leading to abnormal maturation, which was characterized by desynchronization of nuclear and cytoplasmic maturation, along with indications of cytoplasmic degeneration. Immature heterophils appeared in our blood smears, which suggests that the participant birds were coping with active inflammation. Thus, the results showed an immunological stress response in birds, which seems to be associated with the simultaneous on-farm AW assessment. The morphology of heterophils in broiler chickens some days before slaughter is often affected by a number of factors (30). During the production cycle, birds can be subjected to chronic stresses, exposure to infectious agents, and other challenging conditions, which can result in changes in heterophil morphology. These changes often include characteristics such as size, the presence of hyposegmented nuclei, cytoplasmic vacuolation, and abnormal granulations (32), which is compatible with our results.

The higher frequencies of breast and abdominal dermatitis, plumage cleanliness, and lameness found in PH2 and PH4 suggested the worse environmental conditions within these PH as compared with PH1 and PH3. Such results are coherent with the findings as follows: higher percentage of the number heterophils and worse results for abnormalities in heterophil morphology. The higher frequencies of the indicators such as pododermatitis and lameness (health), relative humidity (environmental), percentage of mature and immature heterophils, heterophil morphology, and H:L ratio were found in PH4. Such results may be related to the fact that pododermatitis is an inflammatory process, likely associated with pain and severe lesions. The behavior expressions observed in these birds are also coherent: lethargic, apathetic, bored, frustrated, painful, uneasy, disturbed, scared, and fearful (Figure 3). Weeks et al. (61) observed that birds aged between 39 and 49 days of age remained lying, on average, 76% of the time, and this percentage increased to 86% for birds with a score of 3 for lameness. Sans et al. (6) described that the mean resting time was 55.0% and lameness scores 2 and 3 showed high percentages (82.9%). Deprivation of environmental complexity and incentive may also be a cause of high frequencies of resting behavior. According to Bailie et al. (62), birds may engage in other activities if stimulated.

The QBA allowed the classification of the birds’ behaviors into two distinct groups: from ‘Attentive’ to ‘Desperate’ and from ‘Relaxed’ to ‘Positively occupied’. Heat map (Figure 3) analysis revealed that PH 4 differed from the other farms, with a higher frequency of birds exhibiting behaviors from the first group. This suggests that these birds may be experiencing more stress or unfavorable conditions compared with the birds in the other farms, which displayed less activity related to group 2 behaviors. These results are consistent with the mortality rate, the number of birds in the house on the day of the visit, the changes in health indicators, especially lameness, and finally the percentage of heterophils and cell scores 3–4 for birds of PH 2 and 4.

The assessment of the birds’ health classified various health indicators into different categories based on the severity of their conditions. The results reveal significant differences among the PH, with PH 4 and PH 2 generally scoring worse on the health indicators. Notably, the results for pododermatitis and hock burn were better in the ‘no abnormality’ category (c1) for PH 3 and PH 4.

Physiologically, the high levels of inflammation suggested by the WBC counts and morphological abnormalities can be the result of many factors, such as the response to the release of molecules called damage-associated molecular patterns (DAMPs), as a result of cell death (63, 64). As shown by our results, white cell morphology seems a good biomarker candidate because it is responsive to a variety of factors that impact welfare in our experimental conditions and it has been studied since 1960s (65), with a proportional amount of cumulated knowledge. Additionally, an interesting condition that indicates the choice H/L ratio as a parameter for stress and welfare assessment is its high heritability, which is known since it is used as an indicator for disease resistance in poultry breeding. This is especially important in broiler chickens because it suggests a characteristic that can be selected as a genetic marker, capable of identifying chickens with a higher ability in the immune response, i.e., more immunocompetent chickens (58, 66). As our results showed a relevant potential of heterophil morphological abnormalities as a welfare indicator, the study of its heritability seems warranted.

Overall, our study showed a high proportion of birds suffering from breast and abdominal dermatitis and pododermatitis and lameness with lower plumage cleanliness. All such individual on-farm welfare indicators, showing low bird welfare status, were coherent with the morphologic abnormality of heterophils and heterophil to lymphocyte (H/L) ratios which were observed.

The immunological indicators may represent overall welfare status, i.e., the combination of a multifactorial basis which integrates the average welfare status of animals within a given environmental condition (PH). We wish to know more about WBC as indicators; however, due to the global outbreak of highly pathogenic avian influenza, we have had limited research.

## 5 Conclusion

Stress responses that occur during the rearing and production period of animals induce immunomodulatory and adaptive effects that alter the counts and the morphology of defense cells. The understanding of these phenomena in avian is limited and generally centered on counting leukocytes and heterophil/lymphocyte ratio to measure stress. Our results highlighted the negative impact of environmental and management conditions that existed in each studied PH on the affective states and selected health indicators and WBC profiles of the birds. Targeted interventions are urgently needed to improve the welfare of birds in intensive rearing systems; according to heterophil morphology, broiler chicken welfare in modern PH is critically low. The immunological indicators may represent overall welfare status, i.e., the combination of a multifactorial basis which integrates the average welfare status of animals within a given environmental, health, and behavioral condition.

We also suggest practical considerations and the potential of the morphological characteristics of leukocytes as indicators of welfare, constituting sensitive and low-cost measurements. Indicators which reflect multiple welfare impacts, such as WBC counts and morphological alterations, can become powerful storytellers in the complex task of assessing the welfare of animals, and further research seems warranted as immunological indicators may prove useful as welfare markers both on-field and at the slaughterhouse.

## Data availability statement

The original contributions presented in the study are included in the article/Supplementary material, and further inquiries can be directed to the corresponding author.

## Ethics statement

The animal studies were approved by Animal Use Ethics Committee of the Agricultural Campus (protocol No 023/2022) of the Federal University of Paraná, Curitiba, Brazil. The studies were conducted in accordance with the local legislation and institutional requirements. Written informed consent was obtained from the owners for the participation of their animals in this study. Written informed consent was obtained from the individual(s) for the publication of any potentially identifiable images or data included in this article.

## Author contributions

LR: Conceptualization, Data curation, Formal analysis, Funding acquisition, Investigation, Methodology, Project administration, Resources, Visualization, Writing – original draft, Writing – review & editing. ES: Data curation, Investigation, Methodology, Writing – original draft. RS: Data curation, Writing – review & editing. CT: Formal analysis, Writing – original draft, Writing – review & editing. RF: Data curation, Writing – original draft. CM: Conceptualization, Funding acquisition, Methodology, Supervision, Writing – original draft, Writing – review & editing.
