# Supplementary material for: Will the white blood cells tell? A potential novel tool to assess broiler chicken welfare
Source: Front Vet Sci. 2024 Jul 2;11:1384802. doi: 10.3389/fvets.2024.1384802 (PMC11250086; doi:10.3389/fvets.2024.1384802)
Supplement: Supplementary file 1 [file Data_Sheet_1.pdf]

Supplementary Material

Will the White Blood Cells Tell? A Potential Novel Tool to Assess Broiler Chicken Welfare

Laura Raquel Rios Ribeiro[1*], Elaine Cristina de Oliveira Sans[2]; Ricardo Martins Santos[3]; César Augusto Taconeli[4]; Roberta de Farias[1]; Carla Forte Maiolino Molento[1]

*** Correspondence:** Corresponding Author: laura.ribeiro@ufpr.br

1 Supplementary Data

2 Supplementary Figures and Tables

2.1 Supplementary Figures

Supplementary Figure 1S to 3S.

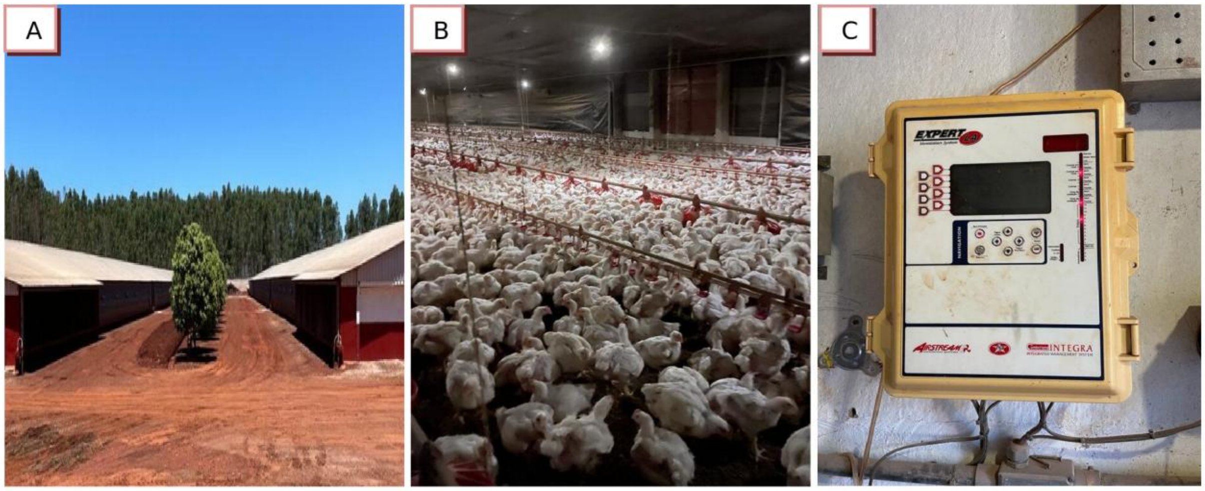

Figure 1S External (A), internal (B) and controller (C) views of poultry farm houses in the Southwest of the State of Mato Grosso do Sul, Brazil.

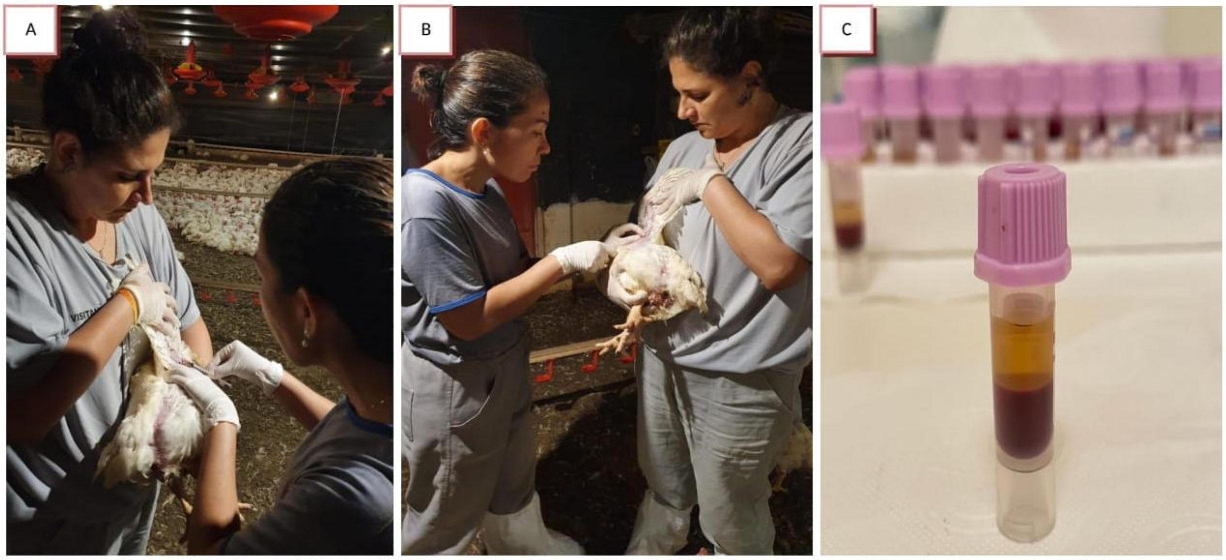

Figure 2S Blood was collected from the wing vein (A-B) in 80 birds from four poultry houses assessed in the Southest of Mato Grosso do Sul, Brazil, in November, 2023, and stored in EDTA tubes (C).

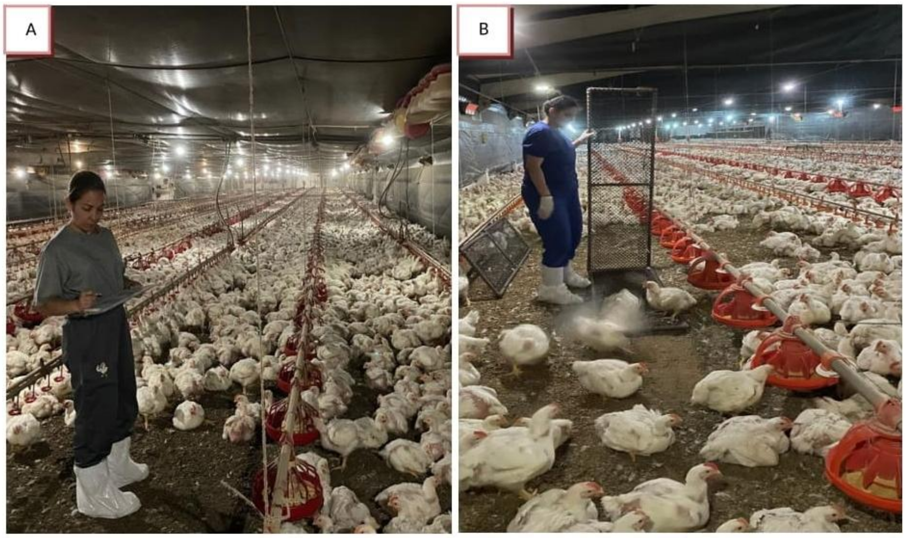

Figure 3S Internal view (A) of poultry house and close-up of groups of birds (B) during Qualitative Behavior Assessment Assessment as per the Welfare Quality® Protocol, Southest of Mato Grosso do Sul, Brazil.

2.2 Supplementary Tables 1S to 5S

Table 1S. Description of the measures[1] of the Welfare Quality assessment protocol for poultry applied in broiler chickens (*N=350 birds assessment per house; total N=1400*) located in the State of Mato Grosso do Sul, Brazil.

Animal welfare measure	Description of the assessment	Sample, birds/house
Breast and abdominal dermatitis	The birds were caught for the examination of breast dermatitis lesion and comparing with a 3 points, where scale zero (absence of lesion) and scale three (severe lesion).	100

Plumage cleanliness	The birds were caught for the examination of plumage cleanliness and comparing with a 3 points, where scale zero (plumage clean) and scale three (plumage very dirty).	
Pododermatitis	The birds were caught for the examination of pododermatitis lesion and comparing with a 5 points, where scale zero (absence of lesion) and scale four (severe lesion).	
Hock burn	The birds were caught for the examination of hock burn lesion and comparing with a 5 points, where scale zero (absence of lesion) and four (severe lesion).	
Lameness	The birds were individually stimulated to walk and a visual inspection of walking ability of the birds was realize and comparing with a 5 points, where scale zero (normal gait) and five (unable to walk).	150
Number of drinkers	The type and number of drinkers and number of housed birds were registered.	-
Density	Barn dimensions (m^2), broiler number and average body weight (kg) were registered at each visit to calculate bird density.	-
Mortality	Number of birds found dead in the barn, according to barn control spreadsheet.	-
Qualitative behavioral assessment	The birds were observed for ten minutes. The group was scored between zero (absence) and 125 (maximum expression) for each positive and negative emotional state.	100

[1] Measures were adapted; for full and original descriptions see Welfare Quality® Assessment Protocol for Poultry (WELFARE QUALITY®, 2009).

Table 2S. Temperature, air velocity, light intensity, relative humidity and ammonia (NH3) assessed poultry houses, Southwest of Mato Grosso do Sul, Brazil.

Variable	PH 1 Itaporã	PH 2 Dourados	PH 3 Glória de Dourados	PH 4 Laguna Caarapã
Outdoor Temperature (ºC)	32.00	30.20	29.50	25.40
Indoor Temperature (ºC)	31.10	28.50	27.00	26.25
Indoor Air velocity (m/s)	2.20	3.20	2.50	2.00
Outdoor Air velocity (m/s)	1.60	3.10	3.60	1.50
Light intensity (lx) indoor	15	15	12	10
Light intensity (lx) outdoor	>20.000	>20.000	>20.000	12810
Indoor Relative Humidity (%)	47.00	55.20	51.00	63.50
Outdoor Relative Humidity (%)	24.70	47.20	45.00	76.00
Ammonia (ppm)	7.50	9.00	8.00	7.00

Table 3S. Classification the scores of the health indicators assessment in poultry houses of poultry houses (PH), Southwest of Mato Grosso do Sul, Brazil.

Health indicator	Classification	PH 1	PH 2	PH 3	PH 4
Breast and abdominal Dermatitis %	C1 (0) - normal	62	17	41	58
	C2 (1) - moderate abnormality	31	38	32	32
	C3 (2 to 3) - severe abnormality	7	45	27	10
Plumage cleanliness %	C1 (0) - normal	39	22	47	41
	C2 (1) - moderate abnormality	53	45	43	41
	C3 (2 to 3) - severe abnormality	8	33	10	18
Pododermatitis%	C1 (0) - normal	78	69	60	32
	C2 (1) - moderate abnormality	14	22	15	12
	C3 (2 to 4) - severe abnormality	8	9	25	56
Hock burn %	C1 (0) - normal	54	70	97	86
	C2 (1) - moderate abnormality	42	23	3	13
	C3 (2 to 4) - severe abnormality	4	7	0	1
Lameness %	C1 (0 to 1) - normal	5	17	0	0
	C2 (2 to 3) - moderate abnormality	81	79	93	50
	C3 (4 to 5) - severe abnormality	13	3	7	50

Table 4S. Hematological parameters and differential cell count from 80 birds in each of the four studied houses, Southwest of Mato Grosso do Sul, Brazil.

Variable	PH 1 Itaporã	PH 2 Dourados	PH 3 Glória de Dourados	PH 4 Laguna Caarapã	Reference Values
Heterophil (%)	35.8 [a]	43.5 [b]	60.3 [c]	60.9	15-50 [1]
Band+Meta+Mielo (%)	0.3	0.1	1.1	1.2	
Lymphocyte (%)	59.2 [a]	50.6 [b]	25.4 [c]	31.0 [d]	29-84 [1]
Eosinophil (%)	2.1	2.7	0.9	1.8	0-16 [1]
Basophil (%)	0.0	0.0	0.0	0.0	0-8 [1]
Monocyte (%)	2.4 [a]	3.1 [a]	12.7 [b]	5.1 [c]	0-7 [1]
Heterophil/Lymphocyte ratio	0.70 [a]	1.03 [b]	3.03 [c]	2.58 [c]	0.2-0.7 [2,3]

[1]Thrall et al. (2015)
[2]Gross & Siegel (1993)
[3]Carpenter & Harms (2023)

Table 5S. Description of the main points of the statistical analysis (response variables, response measure, predictor, experimental unit, statistical modal, statistical tests and model fit assessment).

Response variable	Response Measure	Predictor	Experimental unit	Statistical model	Statistical tests	Model fit assessment
Heterophils	Number of heterophils in 100 blood cells	Poultry houses	Birds	Quasi-binomial GLM	Wald test followed by Tukey's HSD	Residual analysis
Lymphocites	Number of lymphocites in 100 blood cells					
Monocytes	Number of monocytes in 100 blood cells					
HL Ratio	HL Ratio value					
Breast and abdominal dermatits[1]	Visual inspection, comparing with a 4 points scale (percentage of birds scoring 0 to 3)	Poultry houses	Birds	Proportional odds regression model	Likelihood ratio test followed by Tukey's HSD	Residual analysis
Plumage cleanliness[1]	Visual inspection, comparing with a 4 points scale (percentage of birds scoring 0 to 3)					
Pododermatitis[1]	Visual inspection, comparing with a 5 points scale (percentage of birds scoring 0 to 4)					
Hock burns[1]	Visual inspection, comparing with a 5 points scale (percentage of birds scoring 0 to 4)					
Lameness[1]	Visual inspection of walking ability using a 6 points scale (percentage of birds scoring 0 to 5)					
QBA variables	Exploratory analysis using heatmap					

[1]Response variable from Welfare Quality® Assessment Protocol for Poultry
